# Supplementary material for: Aggregation mechanism of colloidal kaolinite in aqueous solutions with electrolyte and surfactants
Source: PLoS One. 2020 Sep 21;15(9):e0238350. doi: 10.1371/journal.pone.0238350 (PMC7505426; doi:10.1371/journal.pone.0238350)
Supplement: S1 File — (DOCX) [file pone.0238350.s001.docx]

Data for Fig. 1:

| pH=3.04 | | pH=6.72 | | pH=10.01 | |
| --- | --- | --- | --- | --- | --- |
| Time (min) | Transmittance (%) | Time (min) | Transmittance (%) | Time (min) | Transmittance (%) |
| 0 | 0 | 0 | 0 | 0 | 0 |
| 4 | 8.6614 | 6 | 0.3874 | 4 | 0.3672 |
| 14 | 23.6992 | 10 | 0.3877 | 12 | 0.374 |
| 22 | 36.7503 | 18 | 0.3887 | 20 | 0.3985 |
| 30 | 41.8111 | 26 | 0.3913 | 28 | 0.4288 |
| 38 | 46.5824 | 34 | 0.3907 | 36 | 0.4595 |
| 46 | 53.0677 | 42 | 0.3922 | 44 | 0.4909 |
| 54 | 54.3607 | 50 | 0.3939 | 52 | 0.5212 |
| 60 | 54.5192 | 56 | 0.3942 | 60 | 0.5508 |
|  |  | 60 | 0.3947 |  |  |

Data for Fig. 2:

| pH | Zeta potential (mV) |
| --- | --- |
| 9.98 | -174.37 |
| 8.99 | -162.12 |
| 8 | -156.92 |
| 7 | -144.59 |
| 5.99 | -130.26 |
| 4.97 | -109.77 |
| 4.05 | -72.18 |
| 3.04 | -13.04 |
| 2.02 | 30.5 |

Data for Fig. 5:

| 0.2 mmol/LNaCl | | 1 mmol/LNaCl | | 3 mmol/LNaCl | | 5 mmol/LNaCl | |
| --- | --- | --- | --- | --- | --- | --- | --- |
| Time (min) | Transmittance (%) | Time (min) | Transmittance (%) | Time (min) | Transmittance (%) | Time (min) | Transmittance (%) |
| 0 | 0 | 0 | 0 | 0 | 0 | 0 | 0 |
| 10 | 0.39111 | 10 | 6.22239 | 10 | 10.3291 | 10 | 12.93841 |
| 20 | 0.39111 | 20 | 11.85033 | 20 | 15.8931 | 20 | 25.16895 |
| 30 | 0.39111 | 30 | 17.85 | 30 | 24.653 | 30 | 37.15 |
| 40 | 0.39111 | 40 | 22.5344 | 40 | 33.421 | 40 | 45.86707 |
| 50 | 0.39111 | 50 | 25.49 | 50 | 38.2371 | 50 | 52.48 |
| 60 | 0.39111 | 60 | 27.41 | 60 | 41.491 | 60 | 56.66 |

| 10 mmol/LNaCl | | 15 mmol/LNaCl | | 20 mmol/LNaCl | |
| --- | --- | --- | --- | --- | --- |
| Time (min) | Transmittance (%) | Time (min) | Transmittance (%) | Time (min) | Transmittance (%) |
| 0 | 0 | 0 | 0 | 0 | 0 |
| 10 | 16.20282 | 10 | 16.18859 | 10 | 16.059 |
| 20 | 31.05 | 20 | 31.46 | 20 | 31.21 |
| 30 | 43.33 | 30 | 44.29 | 30 | 43.91 |
| 40 | 52.06 | 40 | 52.41 | 40 | 52.55 |
| 50 | 58.91 | 50 | 57.82 | 50 | 57.92 |
| 60 | 61.57 | 60 | 60.86 | 60 | 60.67 |

Data for Fig. 6:

| NaCl Concentration (mmol/L) | Zeta potential (mV) |
| --- | --- |
| 0 | -110.09 |
| 1 | -89.81 |
| 3 | -59.42 |
| 5 | -45.12 |
| 10 | -21.01 |
| 15 | -13.89 |
| 20 | -11.59 |

Data for Fig. 7:

Y(x) is V_T_, x is the separation between two particles

0.2 mmol/L NaCl: Y(x) = 1.35$*$exp(-0.0465$*$x)-1.11/x

1 mmol/L NaCl: Y(x) =0.78$*$exp(-0.104$*$x)-1.11/x

5 mmol/L NaCl: Y(x) =0.27$*$exp(-0.2325$*$x)-1.11/x

Data for Fig. 8:

| No surfactant | | DDACl | | SO | |
| --- | --- | --- | --- | --- | --- |
| pH | Transmittance (%) | pH | Transmittance (%) | pH | Transmittance (%) |
| 2.01 | 5.42 | 2.03 | 16.27 | 2 | 17.70686 |
| 2.5 | 6.75 | 2.91 | 16.55 | 3 | 19.6804 |
| 2.98 | 9.55 | 5.01 | 19.27 | 4 | 6.82147 |
| 3.52 | 9.22 | 6.68 | 27.3 | 5 | 0.8421 |
| 4 | 4.82 | 7.99 | 32 | 6.5 | 0.4213 |
| 5.05 | 0.39 | 9.98 | 36.2 | 8 | 0.3231 |
| 6.02 | 0.38 |  |  | 10 | 0.3176 |
| 6.99 | 0.38 |  |  |  |  |
| 8.99 | 0.39 |  |  |  |  |
| 9.99 | 0.39 |  |  |  |  |

Data for Fig. 9:

| DDACl | | SO | |
| --- | --- | --- | --- |
| pH | Zeta potential (mV) | pH | Zeta potential (mV) |
| 9.98 | -104.87 | 9.99 | -179.29 |
| 8.96 | -89.98 | 8.5 | -166.53 |
| 8 | -59.8 | 7.48 | -162.73 |
| 7.01 | -48.54 | 6.47 | -155.78 |
| 5.77 | -31.39 | 5.53 | -141.74 |
| 4.49 | -19.6 | 4.48 | -107.52 |
| 3.55 | -2.17 | 3.52 | -52.25 |
| 2.52 | 20.52 | 2.54 | 10.17 |
| 2.04 | 28.79 | 2.02 | 28.97 |

Data for Fig. 10:

| DDACl | | SO | |
| --- | --- | --- | --- |
| pH | Adsorption density (´10^-10^ mol/cm^2^) | pH | Adsorption density (´10^-10^ mol/cm^2^) |
| 3 | 7.502 | 3 | 25.74 |
| 5 | 16.733 | 4 | 15.18 |
| 5.98 | 19.49 | 5 | 7.56 |
| 8.02 | 23.791 | 6.5 | 5.09 |
| 9.03 | 28.61 | 8 | 2.48 |
| 9.98 | 30.867 | 10 | 1.46 |

Data for Fig. 11:

Y(x) is V_R_, x is the separation between two particles

SO: Y(x) = 0.67$*$exp(-0.0496$*$x)

No surfactant: Y(x) = 0.525$*$exp(-0.0496$*$x)

DDACl: Y(x) = 0.015$*$exp(-0.0496$*$x)

Data for Fig. 12:

Y(x) is V_T_, x is the separation between two particles

SO: Y(x) = 0.67$*$exp(-0.0496$*$x)-1.11/x-5.87$*$exp(-0.155$*$x)

No surfactant: Y(x) = 0.513$*$exp(-0.0496$*$x)-1.11/x

DDACl: Y(x) = 0.015$*$exp(-0.0496$*$x)-1.11/x-5.71$*$exp(-0.155$*$x)
